# Alcohol-Related Hospitalizations Among Adolescents and Young Adults with Type 1 Diabetes in Spain, 2016−2023

**DOI:** 10.3390/jcm14124053

**Published:** 2025-06-08

**Authors:** Lucia Jiménez-Sierra, Ana López-de-Andres, Valentín Hernández-Barrera, Rodrigo Jiménez-Garcia, David Carabantes-Alarcon, Andrés Bodas-Pinedo, Elena Labajo-Gonzalez, José J. Zamorano-León

**Affiliations:** 1 Department of Public Health and Maternal & Child Health, Faculty of Medicine, Universidad Complutense de Madrid, IdISSC, 28040 Madrid, Spain; lujime05@ucm.es (L.J.-S.); dcaraban@ucm.es (D.C.-A.); abodas@ucm.es (A.B.-P.); josejzam@ucm.es (J.J.Z.-L.); 2 Department of Public Health and Maternal & Child Health, Faculty of Pharmacy, Universidad Complutense de Madrid, IdISSC, 28040 Madrid, Spain; anailo04@ucm.es; 3 Preventive Medicine and Public Health Teaching and Research Unit, Health Sciences Faculty, Universidad Rey Juan Carlos, 28922 Madrid, Spain; valentin.hernandez@urjc.es; 4 Department of Legal Medicine, Psychiatry and Pathology, Faculty of Medicine, Universidad Complutense de Madrid, 28040 Madrid, Spain; melabajo@ucm.es

**Keywords:** alcohol, hospital, admissions, type 1 diabetes, diabetic ketoacidosis, drug use, adolescents

## Abstract

**Background/Objectives:** We aim to describe and analyze the clinical characteristics and hospital outcomes of alcohol-related hospital admissions (ARHAs) among adolescents and young adults (12 to 35 years) with type 1 diabetes (T1D) in Spain from 2016 to 2023. We also aim to determine factors associated with admission to the intensive care unit (ICU). **Methods**: A descriptive observational study was carried out based on the Spanish Hospital Discharge Database (SHDD). ICD-10 codes were used to identify ARHAs in T1D patients. Joinpoint regression was used to analyze the time trend, and multivariable logistic regression was conducted to identify predictors of ICU admission. **Results**: We recorded 39,091 discharges with T1D (52.59% females); of these, 1274 (3.26% prevalence) were identified as ARHAs (74.10% males vs. 25.90% females). Joinpoint regression showed a significant increase among females and stable prevalence in males. Females with T1D and ARHAs were younger than males, and more frequently had diabetic ketoacidosis (DKA), mental disorders, and external causes of morbidity and mortality, whereas greater drug use was detected among males. Overall, 20.80% of ARHAs required ICU admission. Predictors of ICU admission included DKA, drug use, hypoglycemia, and female sex (OR: 1.39; 95% CI: 1.02–1.90). **Conclusions**: The frequency of ARHAs in young people with T1D in Spain rose between 2016 and 2023, mainly owing to the increase in the percentage of females requiring ARHA. DKA and drug and tobacco consumption were very common in ARHAs. Admission to the ICU is frequent, increases over time, and is associated with female sex, DKA, hypoglycemia, and drug use.

## 1. Introduction

Alcohol is the most widely consumed psychoactive substance globally. Alcohol consumption is now a major public health concern, and although global consumption appears to have decreased since 2010, the associated health, social, and economic costs remain unacceptably high [1,2,3].

In Spain and other Western countries, the prevalence of harmful alcohol use in adolescents and young adults remains high [1,2,3,4,5,6,7,8]. In Spain, in the year 2024, the highest prevalence of alcohol consumption in the previous month was observed in individuals aged 15–24 years (10.7% of males and 7.2% of females), decreasing slightly to 10.3% and 4.7%, respectively, in those aged 25–34 years [4].

A particularly alarming trend is the rise in binge drinking. According to the 2023 ESTUDES study, among individuals aged 15 to 19 in Spain, the prevalence of binge drinking in the previous month was approximately 17–18% for both sexes and, in many cases, was associated with the use of other psychoactive substances, mainly tobacco and cannabis [2,3,4,5].

Among adolescents, alcohol misuse increases the risk of injuries, assaults (as either victim or perpetrator), and accidents. It is also linked to high-risk behaviors such as driving under the influence, interpersonal violence, unprotected sexual practices, and drug use [1,2,3,4,5,6,7,8]. In Spain, as in many other countries, these behaviors lead to a high number of emergency room (ER) visits and hospital admissions [1,3,6,7,8].

Despite these well-documented risks, cultural norms often frame alcohol consumption as socially acceptable, leading it to become widespread, even among adolescents with chronic conditions [3,9,10].

In Spain, the prevalence of T1D is around 2 per 1000 inhabitants, corresponding to roughly 100,000 individuals living with the condition with an estimated incidence of 8 new cases per 100,000 inhabitants per year [11,12,13,14,15,16].

Many studies have investigated alcohol use patterns among individuals with T1D and associated factors [17,18,19,20,21,22,23,24,25,26]. Most findings indicate that adolescents and young adults with T1D consume alcohol at rates similar to those of their non-diabetic peers [17,18,19,20,21,22,23]. Male sex, the use of tobacco and other psychoactive substances, and lower engagement with diabetes self-care have all been associated with higher alcohol consumption in young people with T1D [17,18,19,20,21,22,23].

Psychosocial stressors, depressive symptoms, and the desire to fit in socially, despite the illness, may contribute to risky behaviors such as excessive alcohol consumption among young people with T1D [17,18,19,20,21,22,23]. The few Spanish studies conducted on this topic, which are based on small samples, suggest a slightly lower prevalence of alcohol use among adolescents and young adults with T1D than in the general population [13,26].

Alcohol consumption among adolescents and young adults with T1D poses unique metabolic risks. The most common is delayed hypoglycemia, typically occurring 6–8 h after drinking, owing to hepatic prioritization of alcohol metabolism and inhibition of gluconeogenesis [25,27,28]. Moreover, alcohol can mask hypoglycemia symptoms (e.g., confusion, poor coordination), which may be mistaken for intoxication, leading to delays in recognition and treatment and increasing the risk of ER visits, hospitalizations, and fatal outcomes [17,23,25,27,28].

Several investigations have also reported that erratic insulin administration, dehydration, and vomiting induced by excessive alcohol intake can increase the risk of diabetic ketoacidosis (DKA) [9,18,29,30]. The concomitant use of illicit substances can also raise stress hormone levels, further exacerbating the risk of hyperglycemia and DKA [9,22,25,30,31,32].

Common risky behaviors, such as reduced blood glucose monitoring, poor insulin management, and inadequate nutrition, may amplify the harmful effects of alcohol in individuals with diabetes. Even low levels of alcohol intake have been associated with poorer diabetes self-care and decreased attendance at screening for diabetes complications [33,34].

Studies in northern Europe have shown that, within two to three decades of a diagnosis of T1D, alcohol-related causes accounted for a significant proportion of deaths, often linked to episodes of DKA and hypoglycemia [35,36].

We limited our investigation to adolescents and young adults for several reasons. First, many of the problems derived from excessive alcohol consumption are concentrated in this age group, both globally and in Spain [1,2,3,4,5,6,7,8]. Second, in other international studies, adolescents are grouped together with young adults for the study of alcohol consumption, in both the general population and among subjects with T1D [17,19,20,21,24]. Third, the prevalence of type 2 diabetes (T2D) in Spain among those below 35 years of age is very low, which minimizes the possible bias of misclassification of subjects with T2D as T1D [12]. Finally, to our knowledge, few studies have examined alcohol-related hospital admissions (ARHAs) in adolescents and young adults with T1D [17,21], and specific data among young people with T1D are lacking in Spain.

The objectives of this study were as follows: (1) to characterize the clinical profiles and outcomes of ARHAs among adolescents and young adults with T1D, including trends from 2016 to 2023; (2) to identify variables associated with an increased risk of ARHAs; and (3) to determine predictors of severe hospital outcomes following ARHAs.

## 2. Materials and Methods

We performed a descriptive observational study based on the Spanish Hospital Discharge Database (SHDD). The SHDD is a mandatory database managed by the Spanish Ministry of Health (SMH) that collects information on all hospital discharges that occur in Spain. It includes individuals who are admitted to hospitalization wards, and no data are collected from those who are treated only in ERs. The database includes age, sex, dates of admission and discharge, reason for discharge (cure, transfer to another center, or death in the hospital), main diagnosis and up to 19 secondary diagnoses, up to 20 diagnostic or therapeutic procedures performed during admission, and admission to the intensive care unit (ICU). The International Classification of Diseases, Tenth Revision (ICD-10) is used to code diagnoses and procedures. Detailed information on the SHDD can be found elsewhere [37].

We analyzed the records of all discharges from Spanish public hospitals from 1 January 2016 to 31 December 2023.

The study population comprised individuals aged between 12 and 35 years with an ICD-10 code for T1D (E10) in any of the 20 diagnostic fields. Those with a code for type 2 diabetes (T2D) (ICD-10 E11) were excluded, as were those whose sex, dates of admission and/or discharge, or reason for admission had not been collected.

The main study variable was ARHA. ICD-10 codes that define ARHA were adopted from Trefan et al. and included E24.4; E51.2; F10; G31.2; G62.1; G72.1; I42.6; K29.20; K29.21 K70; K85.2; K86.0; T51.0; O35.4; R78.0; Y90; and Z71.4 [8]. Code T51.9 (Toxic effect: Alcohol, unspecified) was not used to define ARHA in our investigation because it was not selected by Trefan et al. [8]. Codes X45 (Accidental poisoning by and exposure to alcohol) and Y91.x (Evidence of alcohol involvement determined by level of intoxication) could not be used because these codes are not included in the Spanish version of the ICD-10 applied in the SHDD https://www.eciemaps.sanidad.gob.es/browser/metabuscador (accessed on 4 May 2025).

ARHA was defined by the presence of any of these codes in any of the 20 diagnostic coding positions [17]. In under 5% of cases, the codes for ARHA appeared as the primary diagnosis; therefore, no subgroups analysis was performed considering the diagnosis position.

The analysis was stratified by sex. The covariates included year of admission, age (as continuous and in groups), and characteristics and outcomes of hospitalization, such as weekend admissions (including Saturday or Sunday), ICU admission, length of hospital stay (LOHS, calculated as the difference between discharge and admission date), and in-hospital mortality (IHM).

The clinical conditions analyzed as diagnoses coded in any position on the discharge reports included DKA, hypoglycemia, mental disorders, drug use, tobacco use, external causes of morbidity and mortality, and COVID-19. The ICD-10 codes that were used to define these study variables are detailed in Table S1. There were no coding changes for any of the study variables (besides the inclusion of the ICD-10 code for COVID-19) over the study period.

### 2.1. Statistical Analysis

The study population was described by years and study variables using means and standard deviations for quantitative measures that were normally distributed (Kolmogorov–Smirnov test) and median and interquartile range (IQR) for variables that were not (LOHS). Qualitative variables were expressed as counts and percentages.

Changes from 2016 to 2023 in the prevalence of ARHAs over total admissions with a diagnosis of T1D in males and females were analyzed using the Joinpoint Regression Program, which enables us to test whether an apparent change in trend is statistically significant based on the annual percent change [38].

For the remaining covariates, the temporal trend was assessed using the linear regression *t*-test or Jonckheere–Terpstra test (LOHS) for quantitative variables and the Cochran–Mantel–Haenszel statistic for qualitative variables.

In the bivariable analysis, normally distributed quantitative variables were compared using the *t*-test (means); non-normally distributed variables were compared using the Mann–Whitney test (medians) (LOHS). The Fisher exact test was used to compare percentages. The Bonferroni–Holm method was applied to adjust *p*-values for multiple testing when necessary.

Multivariable unconditional logistic regression was conducted using a generalized lineal model to identify which study variables were associated with ARHAs in children and young adults with T1D in Spain from 2016 to 2023.

This same multivariable method was also applied to identify which covariates were associated with T1D and ARHA in a patient with a severe condition and requiring admission to the ICU. All multivariable modeling was performed separately for each gender.

All independent variables with a significant bivariate association (*p* < 0.1) and those considered scientifically relevant, including potential confounders based on the reviewed literature, were included in the initial model. For all models, we included the following variables: age groups, weekend admissions, DKA, hypoglycemia, mental disorders, drug use, tobacco use, external causes of morbidity and mortality, COVID-19, year of admission, and gender. The importance of each independent variable was assessed using the Wald statistic, and variables that did not contribute significantly were eliminated through an iterative process, comparing models with the Likelihood Ratio test. Collinearity was evaluated using the Variance Inflation Factor (VIF), and potential two-way interactions were tested. Model fit was assessed using the Hosmer–Lemeshow test. We provided the odds ratio (OR) with its 95% confidence interval (CI) as a measure of association.

We followed the methodology for the construction of the models described by Hosmer et al. [39].

The descriptive and statistical analyses were performed with Stata version 14 (Stata, College Station, TX, USA). Statistical significance was set at *p* < 0.05 (2-tailed).

### 2.2. Ethical Aspects

The SHDD is an administrative database owned by the SMH. To obtain the data, a request indicating the objectives and methodology of the proposed research must be submitted [40]. The SMH assesses the request from a scientific and ethical point of view and decides whether it is appropriate to send the data. The data received to carry out this research were fully anonymized and were provided free of charge. For all of the above, and in accordance with Spanish legislation, the authorization of a clinical trial committee is not required [41].

## 3. Results

Between 2016 and 2023 in Spain, the total number of hospital discharges among persons aged between 12 and 35 years with a diagnosis of T1D in any position amounted to 97,070. After applying the selection criteria, 39,091 discharges were identified, 18,533 (47.41%) corresponding to males and 20,558 to females (52.59%).

In 1274 adolescents and young adults with T1D (3.26%), an ARHA code had been entered at any diagnostic position. We observed a significantly higher prevalence in males (5.09%) than in females (1.61%) (*p* < 0.001).

**Figure 1:**
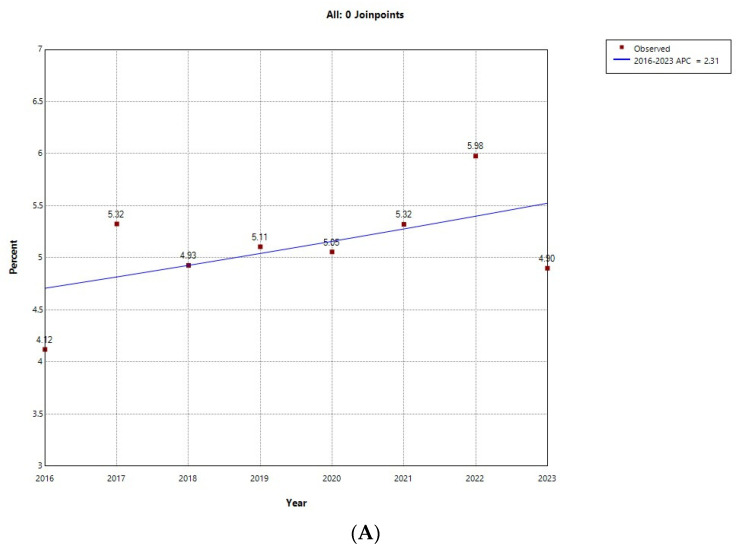

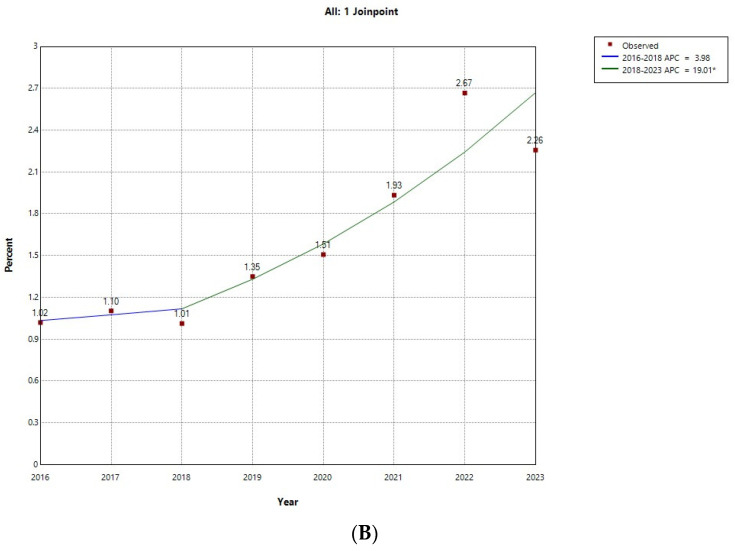
Joinpoint regression for the prevalence of alcohol-related hospital admission over all type 1 diabetes hospital admissions in (**A**) male; (**B**) female children and young adults in Spain (2016−2023). * Indicates that the Annual Percent Change (APC) is significantly different from zero at the alpha = 0.05 level. Final Selected Model: 0 Joinpoints.

Figure 1 shows the results of the Joinpoint regression analysis for the prevalence of ARHAs among admissions with T1D for males (Figure 1A) and females (Figure 1B). Among males, prevalence did not vary significantly, ranging from 4.12% in 2016 to 5.98% in 2022. Among females, prevalence remained stable in the period 2016 to 2018, with values around 1%, and grew with an annual percent change of 19.01% between 2019 and 2023, peaking in 2022 with a prevalence of 2.67%.

Table 1 shows the number, sex, age, and hospital outcomes of ARHAs in children and young adults with T1D in Spain between 2016 and 2023.

In absolute numbers, there was an increasing trend in ARHAs, increasing from 119 in 2016 to 211 in 2022, with this increase being more evident among females. In all the years of the study period, the percentage of males was higher than that of females, although the percentage of females increased significantly from representing 21.85% of admissions in the first year analyzed to 32.77% in the last year (*p* = 0.008). The mean age of the ARHA patients did not vary, being around 27 years. By age group, it was observed that every year, most cases occurred in the older age range (30–35 years) and that the 12- to 17-year-old group accounted for the smallest percentage of ARHAs, with no significant changes over time.

The proportion of ARHAs that occurred on weekends did not change significantly (*p* = 0.565) between 2016 and 2023, with a maximum of 29.45% in 2019, a minimum of 20.85% in 2023, and an overall value of 26.14%.

Among ARHAs, the frequency of admission to the ICU for the entire period was 20.80%, increasing from 13.45% in 2016 to 25.42% in 2023 (*p* = 0.009). The median LOHS remained stable at around 4 days, and only one IHM occurred during the period studied.

Table 2 shows the clinical characteristics of ARHAs in children and young adults with T1D in Spain between 2016 and 2023.

DKA had been coded in 771 of the ARHAs (60.52%), with no significant changes in prevalence during the period studied. The prevalence of hypoglycemia ranged from 3.32% in 2022 to 7.64% in 2017, although the difference was not significant. The presence of mental disorders did not vary significantly either, with the value for the entire period being 15.46%.

Drug use was coded in 49.69% of all ARHAs in the 8 years analyzed, with the prevalence ranging from 41.72% in 2019 to 54% in 2020 (non-significant).

During the study period, more than half of the ARHAs had a tobacco use code, with the highest figures in 2020 (69.33%) and the lowest in 2023 (52.54%), although the variation over time did not reach statistical significance.

A code for external causes of morbidity and mortality appeared in the discharge report in 11.07% of the ARHAs (range: 3.6–15.33%; *p* for time trend 0.065).

Only 25 (3.53%) of the 709 ARHAs involving children and young adults admitted between 2020 and 2023 had a code for COVID-19.

After stratifying the study population by sex, we obtained the results shown in Table 3. Females with T1D and ARHA were younger than males (mean age of 25.31 years vs. 27.88 years; *p* < 0.001), with a higher percentage of adolescents and younger adults (12–17 years, 10.61% vs. 4.56%, and 18–23 years, 33.03% vs. 20.23%). A significantly higher prevalence of ICU admission (25.45% vs. 19.17%; *p* = 0.016), DKA (66.36% vs. 58.47%; *p* = 0.012), mental disorders (25.45% vs. 11.97%; *p* < 0.001), and external causes of morbidity and mortality (15.7% vs. 9.43%; *p* = 0.002) was also found among females. However, males more frequently used drugs (52.12% vs. 42.76%; *p* < 0.001).

Table S2 compares the distribution of study variables in adolescents and young adults with T1D with and without ARHA according to sex. The mean age was significantly higher in ARHA patients of both sexes than in admission patients without ARHAs (males: 27.88 years vs. 22.75 years, *p* < 0.001; females: 25.31 years vs. 23.8 years, *p* < 0.001).

Moreover, a significantly higher proportion of admissions on weekends (males: 25.64% vs. 21.12%, *p* < 0.001; females: 27.58% vs. 20.11%, *p* < 0.001) and to the ICU (males: 19.17% vs. 11.33%, *p* < 0.001; females: 25.45% vs. 12.46%, *p* < 0.001) was observed in both genders with ARHAs.

The prevalence of DKA, mental disorders, drug use, tobacco use, and external causes of morbidity and mortality was also much higher in males and females with ARHA codes than in males and females with T1D without ARHA codes.

Table 4 shows the results of the multivariable analysis, which revealed the study variables associated with having an ARHA code for males, females, and the entire T1D population hospitalized between 2016 and 2023. In all three models, DKA, drug use, and tobacco use were significantly associated with ARHA. Drug use was the variable with the highest ORs, specifically 6.15 (95% CI: 5.27–7.18), 7.01 (95% CI: 5.32–9.21), and 6.40 (95% CI: 5.59–7.33) for males, females, and both sexes, respectively.

Among males, being older and hypoglycemia were significantly associated with the presence of ARHA. Among females, the 18- to 23-year-old age group (compared with the reference group [12–17 years]) and weekend admission were associated with ARHA. For males, the multivariable analysis coincided with the Joinpoint regression by showing positive and significant ORs in the last 3 years compared with the first.

After adjusting for possible confounders, it was observed that among adolescents and young adults with T1D, the probability of ARHA was 2.46 (95% CI: 2.15–2.82) times higher in males than in females.

Table 5 shows the study variables associated with admission to the ICU in children and young adults with T1D and ARHA in Spain according to sex. DKA was the variable most strongly associated with admission to the ICU, with an OR of 4.91 (95% CI: 3.12–7.72) in males, 8.47 (95% CI: 3.61–19.86) in females, and 5.59 (95% CI: 3.78–8.26) in both sexes. A code for drug use was also significantly associated with ICU admission in all three populations. However, hypoglycemia was associated in males (OR: 4.11; 95% CI: 2–8.47) but not in females. Age was not associated with admission to the ICU in males, females, or both sexes, although the OR for females (3.87; 95% CI: 1.03–15.03) and both sexes (OR: 2.08; 95% CI: 1.07–4.09) for the year 2023 was significantly higher than for 2016.

When the entire study population was analyzed, it was found that females with T1D and ARHA were 39% more likely to require admission to the ICU than males (OR: 1.39; 95% CI: 1.02–1.90).

## 4. Discussion

The most relevant results of this study are the increase in the number of ARHAs among adolescents and young adults with T1D in Spain between 2016 and 2023, mainly due to the increase in females requiring ARHA. Overall, males still represent three quarters of cases, but the percentage of females rose from 21.85% in 2016 to 32.77% in 2023. The prevalences of DKA, drug use, and tobacco use were very high among ARHAs. Severe cases, which required admission to the ICU, were frequent and associated with female sex, DKA, hypoglycemia, and drug use.

ER visits for the consumption of alcohol and other psychoactive substances in the general Spanish population increased significantly between 2016 and 2021, with a higher percentage of males requiring ER visits [2,3]. This greater frequency of ER visits and hospitalizations for alcohol-related damage among males has also been observed in other countries, where, as in Spain and in our investigation, the gap between the sexes is narrowing [6,7,42,43,44,45,46,47].

Analysis of young people with T1D up to 37 years of age in Wales revealed that females were overall 20% less likely to require ARHA than males [17].

The DKA code was recorded in 60.52% of the ARHAs in our study. DKA is a common acute complication that causes a very significant number of ER visits and hospitalizations in young people with T1D [18,29,30,48,49,50,51,52]. We found the prevalence of DKA among ARHAs to be significantly higher in females than in males. These sex differences have been pointed out by other authors [30,49,50,51,52] and are believed to result from the higher prevalence among females of psychiatric or eating disorders (e.g., patients omitting insulin to induce weight loss) and psychological and societal pressures such as peer pressure, body image, and risk-taking behavior [48,49,50,51,52,53].

Alcohol consumption as a risk factor for DKA in young people with T1D has been reported [9,18,23,25,29,30].

In Spain, Isidro et al. analyzed 253 episodes of diabetic ketosis and DKA among patients admitted to the hospital, of which 93.1% occurred in T1D patients, finding that 20.6% were related to substance use. Cocaine, followed by cannabis and alcohol, was the most frequently involved substance [9]. An association between illicit drug use and hospital admission among adolescents and adults with T1D has been reported [9,20,31,32,54,55].

In our study population, half of the ARHAs had a code for drug use. The proportions for cannabis and cocaine were 70.46% and 50.41%, respectively, and in 27.80%, both drugs were codified in the same patient. In Spain, in 2021, the most frequent illicit drug related to ER visits in the general population was cannabis (44.5%), followed by cocaine (40.3%). By age and sex, both substances are detected more frequently in males than in females [2,4,5]. Our results coincide with those of the general population, identifying greater drug use among males with T1D than among females.

A recent review found an increased risk of DKA among individuals with T1D who use cannabis [54].

The association between cocaine and hospital admissions for DKA has been well reported, being one of the most frequently identified illicit drugs in hospitalized T1D patients [9,25,56]. Concomitant use of alcohol and illicit drugs has an additive mind-altering effect, which could further impair an individual’s ability to adequately manage their diabetes [25].

The overall proportion with a code for tobacco use in the study population amounted to 63.19%, remaining unchanged between 2016 and 2023, and with no differences by sex. Smoking status has been closely related to alcohol consumption among people with T1D [18,19,23,57]. As smoking is known to be associated with inadequate glycemic control, it could also contribute to an increased risk of DKA events in alcohol users [18,57,58,59].

The association between psychiatric morbidity (depression, anxiety, or eating disorders) and hospitalizations has been reported more frequently in females than in males with T1D [17,29,30,60,61,62,63,64]. A quarter of young female ARHAs in our study had a mental disorder code, with the percentage falling to less than half (11.97%) among males.

Evidence shows that in the general population, levels of anxiety and depression are increasing among adolescents, particularly females, and that this increase may have accelerated since the COVID-19 pandemic [65,66]. Previous studies have suggested that females are more likely than males to consume alcohol to cope with mental disorders [43,67].

The prevalence of external causes of morbidity and mortality among ARHAs in our study was 11.07%. The association between external causes and ARHA, mainly accidents, falls, and intentional self-harm, is frequent in adolescents and young people in the general population [6,8]. An association between the risk of falls and alcohol consumption in individuals with T1D has been reported [68].

Combining alcohol consumption with other risk behaviors such as drug use, inadequate glycemic control, or driving under the influence of psychotropic substances can possibly explain the external causes of ARHA among adolescents and young adults with T1D [1,2,3,6,8,68].

During the study period, 20.80% of the young people included in our sample required admission to the ICU, with an increasing trend from 2016 to 2023. In our opinion, possible explanations such as changes in the amount and type of alcohol, the use of concomitant illicit drugs, and the increment in binge drinking in Spain could justify this time trend [2,3,4,5]. Future studies with more detailed data should confirm these hypotheses.

The predictors of admission to the ICU were, in order of magnitude, DKA, hypoglycemia, female sex, and drug use.

The proportion of ARHAs requiring ICU admission is much higher in young people with T1D than in those of the same age range without this disease [6,69,70].

The strong association between DKA and ICU admission is expected since this metabolic disorder is one of the main causes of severe disease and death in persons with T1D [9,30,48,49,71,72,73,74].

The presence of hypoglycemia codes in our population was very low (<5%), and this could be partly due to inadequate reporting. However, in the multivariable analysis, it was significantly associated with admission to the ICU. In the SEARCH for Diabetes in Youth Study, of 602 young T1D patients (mean age of 21.3 years), self-reported severe hypoglycemia was recorded in 12.7% of those who engaged in binge drinking, 9.0% of non-binge drinkers, and 6.7% of non-drinkers [20].

In our study, from 2016 to 2023, admission to the ICU was codified in 181 of 944 (19.17%) males and 84 of 330 (25.45%) females (*p* = 0.016), resulting in a 39% higher risk among females after multivariable adjustment (OR: 1.39; 95% CI: 1.02–1.90). Consistent with our findings, other authors found female sex to be associated with a higher frequency of severe DKA or admission to the ICU, both in newly diagnosed T1D patients and in those with a previous diagnosis [30,75], possibly because the metabolism of alcohol in females means that the same amount affects them more than males [43,76].

The association between drug use and the severity of DKA detected in our study coincides with the conclusions of other authors [9,30].

The reasons for alcohol consumption in individuals with T1D proposed in the literature lead to poor glycemic control and may explain ARHAs. The decrease in psychological well-being and the increased stress associated with T1D likely play an important role in the development of substance use disorders [23]. In addition, as alcohol consumption increases, self-care in people with diabetes decreases [34]. Qualitative studies have indicated that messages about the effect of consuming alcohol and other substances, as well as the management of alcohol and substance abuse, are not always adequately received and accepted among young people with T1D [21]. In fact, in Spain, both alcohol and cannabis consumption are currently “socially acceptable” leisure activities [2,3,9,77].

To reduce and prevent the complications of diabetes caused by alcohol use, adolescents and young adults with T1D should be educated about consuming alcohol responsibly. To this end, young people should be trained to identify symptoms of alcohol intoxication, maintain blood glucose levels within the recommended range, avoid consuming large amounts of alcohol and binge drinking, and ingest carbohydrates while drinking [17,18,19,20,21,22,23,24,25].

New technologies for the treatment of T1D, such as continuous glucose monitoring and insulin pump therapy, should complement educational interventions to improve glucose control during alcohol consumption [78].

Current educational interventions should be reviewed and, where possible, personalized according to sex, age, socioeconomic/educational status, and background. It is essential for healthcare professionals to monitor their T1D patients so that they can detect the consumption of alcohol and other psychoactive substances early [17,18,19,20,21,22,23,24,25].

Among adolescents in particular, the conflicting interests of sociability and the reduction in risk behaviors should be taken into consideration [17,18,19,20,21,22,23,24,25].

More quantitative and qualitative studies exploring the factors that drive risky alcohol and drug use in young people with T1D are needed in order to improve educational strategies and make them more effective. Ultimately, there is a critical need to continue to develop robust, multidisciplinary support structures for adolescents and young adults who face the daily challenges of managing T1D [17,18,19,20,21,22,23,24,25].

The main strength of our study is its population-based approach, which used a stable, exhaustive, valid, and reliable database over a period of 8 years. Therefore, we identified sufficient ARHAs to be able to stratify by both demographic and clinical variables and to build robust and accurate multivariable models. Of note, the SHDD is periodically evaluated to ensure its quality and has been used in previous epidemiological studies [37,46].

Our study methodology is subject to a series of limitations. Individuals with T1D who were admitted more than once during the study period were included in the analysis to facilitate comparison of mean admission rates. Furthermore, given the obligatory confidentiality of the database, it is not possible to identify those people who were admitted more than once. Previous studies have shown that recurrent DKA is frequent in T1D patients [9,29,30,48,50,53,56,60]. As our data were derived from discharge reports, we were dependent on the notes of healthcare providers. This issue could result in information bias due to missing data regarding the context of alcohol intoxication. The possible effect that the COVID-19 pandemic had on hospital admission protocols or coding habits should also be considered.

To our knowledge, the validity of alcohol consumption codes has not been evaluated to date in our database. Other international studies have used databases of hospital discharges to investigate ARHAs [8,79]. In any case, no information is collected on the type or amount of alcohol consumed or in the blood. Regarding T1D codes, a previous Spanish study concluded that ICD codes for diabetes from hospital discharge databases were valid for epidemiological investigations [80]. However, we lack information on the clinical characteristics of T1D, such as duration, treatment modalities (Intensified Conventional Insulin Therapy or insulin pump), compliance, use of new technologies, and HbA1c data. Similarly, we have no data on social or educational levels or the urban or rural settings that have been associated with ARHA in other populations [8,17]. The generalizability of our findings to other populations depends on similarities in health systems, drinking culture, and socioeconomic variables. Unfortunately, in Spain, the national legislation on drug addiction and alcohol consumption has not been modified for more than 10 years, as has been pointed out before by other authors [81]. Therefore, we cannot assess what changes in Spain’s policies on alcohol and addictive drugs over the past few years might have influenced alcohol consumption issues among young people with T1D.

Finally, as the SHDD is an administrative database, it is not designed to prove causal relationships.

## 5. Conclusions

We conclude that alcohol consumption poses a high risk of harm to adolescents and young adults with T1D. ARHAs in young people with T1D in Spain increased between 2016 and 2023, mainly owing to the increase in females requiring ARHA. DKA, drug use, and tobacco consumption were very frequent among ARHAs, with differences in frequency between males and females. Admission to the ICU was frequent, increasing over time and associated with female sex, DKA, hypoglycemia, and drug use. It is necessary to review interventions to reduce the consumption of alcohol and other psychotropic substances in young people with T1D and to deepen our knowledge of the social and psychological factors that favor consumption.

## Figures and Tables

**Table 1:** Number, gender, age, and hospital outcomes of alcohol-related hospital admission in children and young adults with type 1 diabetes in Spain (2016−2023).

		2016	2017	2018	2019	2020	2021	2022	2023	*p* for Trend	Total
		*N (%)*	*N (%)*	*N (%)*	*N (%)*	*N (%)*	*N (%)*	*N (%)*	*N (%)*		*N (%)*
Number of T1D hospital admissions		4806	4718	4938	5210	4596	4860	4964	4999	NA	39,091
Number of ARHAs		119 (2.48)	144 (3.05)	139 (2.81)	163 (3.13)	150 (3.26)	171 (3.52)	211 (4.25)	177 (3.54)	<0.001	1274 (3.26)
Gender	Male	93 (78.15)	116 (80.56)	112 (80.58)	126 (77.3)	115 (76.67)	121 (70.76)	142 (67.3)	119 (67.23)	0.008	944 (74.10)
	Female	26 (21.85)	28 (19.44)	27 (19.42)	37 (22.7)	35 (23.33)	50 (29.24)	69 (32.7)	58 (32.77)		330 (25.90)
Age in years. Mean (SD)		27.87 (5.76)	28.1 (5.73)	26.66 (5.66)	26.82 (6.33)	27.27 (6.19)	27.01 (5.81)	27.41 (5.99)	26.78 (5.95)	0.338	27.22 (5.95)
Age groups	12–17 years	6 (5.04)	6 (4.17)	8 (5.76)	12 (7.36)	9 (6)	8 (4.68)	16 (7.58)	13 (7.34)	0.265	78 (6.12)
	18–23 years	24 (20.17)	30 (20.83)	33 (23.74)	44 (26.99)	44 (29.33)	44 (25.73)	37 (17.54)	44 (24.86)		300 (23.55)
	24–29 years	34 (28.57)	38 (26.39)	46 (33.09)	35 (21.47)	29 (19.33)	50 (29.24)	70 (33.18)	50 (28.25)		352 (27.63)
	30–35 years	55 (46.22)	70 (48.61)	52 (37.41)	72 (44.17)	68 (45.33)	69 (40.35)	88 (41.71)	70 (39.55)		544 (42.7)
Weekend admissions		34 (28.57)	34 (23.61)	40 (28.78)	48 (29.45)	40 (26.67)	49 (28.65)	51 (24.17)	37 (20.90)	0.565	333 (26.14)
Admission to ICU		16 (13.45)	21 (14.58)	43 (30.94)	32 (19.63)	32 (21.33)	32 (18.71)	44 (20.85)	45 (25.42)	0.009	265 (20.80)
LOHS. Median (IQR)		4 (4)	4 (4)	3 (3)	3 (4)	4 (4)	4 (4)	4 (5)	3 (4)	0.213	4 (4)
IHM		0 (0)	0 (0)	1 (0.72)	0 (0)	0 (0)	0 (0)	0 (0)	0 (0)	NA	1 (0.08)

T1D: type 1 diabetes. ARHA: alcohol-related hospital admission. Weekend admissions included Saturday or Sunday. ICU: intensive care unit. LOHS: length of hospital stay. IHM: in-hospital mortality. *p*-Value for time trend calculated using the linear regression *t*-test or Jonckheere–Terpstra test (LOHS) for quantitative variables and the Cochran–Mantel–Haenszel statistic for qualitative variables. NA: not available.

**Table 2:** Clinical characteristics of alcohol-related hospital admission (ARHA) in children and young adults with type 1 diabetes in Spain (2016−2023).

	2016	2017	2018	2019	2020	2021	2022	2023	*p*	Total
	*N (%)*	*N (%)*	*N (%)*	*N (%)*	*N (%)*	*N (%)*	*N (%)*	*N (%)*		*N (%)*
Diabetic ketoacidosis	72 (60.5)	79 (54.86)	100 (71.94)	95 (58.28)	94 (62.67)	107 (62.57)	117 (55.45)	107 (60.45)	0.079	771 (60.52)
Hypoglycemia	6 (5.04)	11 (7.64)	5 (3.60)	7 (4.29)	10 (6.67)	8 (4.68)	7 (3.32)	6 (3.39)	0.527	60 (4.71)
Mental disorders	15 (12.61)	23 (15.97)	16 (11.51)	21 (12.88)	27 (18)	26 (15.2)	37 (17.54)	32 (18.08)	0.584	197 (15.46)
Drug use	54 (45.38)	68 (47.22)	71 (51.08)	68 (41.72)	81 (54)	92 (53.8)	112 (53.08)	87 (49.15)	0.271	633 (49.69)
Tobacco use	72 (60.5)	95 (65.97)	93 (66.91)	100 (61.35)	104 (69.33)	110 (64.33)	138 (65.4)	93 (52.54)	0.061	805 (63.19)
External causes of morbidity and mortality	13 (10.92)	15 (10.42)	5 (3.6)	14 (8.59)	23 (15.33)	22 (12.87)	26 (12.32)	23 (12.99)	0.065	141 (11.07)
COVID-19	0 (0)	0 (0)	0 (0)	0 (0)	0 (0)	6 (3.51)	15 (7.11)	4 (2.26)	<0.001	25 (3.53) *

Mental disorders included ICD-10 codes for depression, anxiety, and specific personality disorders (see Table S1). Drug use included ICD-10 codes for mental and behavioral disorders due to the use of opioids, cannabinoids, sedatives or hypnotics, cocaine, and other stimulants, including caffeine, hallucinogens, volatile solvents, and other psychoactive substances (see Table S1). External causes included ICD-10 codes for accidents, injury, and intentional self-harm (see Table S1). *p*-Value for time trend calculated using the linear regression *t*-test or Jonckheere–Terpstra test (LOHS) for quantitative variables and the Cochran–Mantel–Haenszel statistic for qualitative variables. NA: not available. * Calculated for cases from 2020 to 2023.

**Table 3:** Clinical characteristics and hospital outcomes of alcohol-related hospital admission (ARHA) in children and young adults with type 1 diabetes in Spain according to gender (2016−2023).

		Male	Female	*p*
		*N (%)*	*N (%)*	
Number of T1D hospital admissions		18,533	20,558	NA
Number of ARHAs		944 (5.09)	330 (1.61)	<0.001
Age	Mean (SD)	27.88 (5.66)	25.31 (6.34)	<0.001
Age	12–17 years	43 (4.56)	35 (10.61)	<0.001
	18–23 years	191 (20.23)	109 (33.03)	
	24–29 years	262 (27.75)	90 (27.27)	
	30–35 years	448 (47.46)	96 (29.09)	
Weekend		242 (25.64)	91 (27.58)	0.490
Admission to ICU		181 (19.17)	84 (25.45)	0.016
LOHS. Median (IQR)		3 (4)	4 (4)	0.342
IHM		1 (0.11)	0 (0)	0.554
Diabetic ketoacidosis		552 (58.47)	219 (66.36)	0.012
Hypoglycemia		47 (4.98)	13 (3.94)	0.443
Mental disorders		113 (11.97)	84 (25.45)	<0.001
Drug use		492 (52.12)	141 (42.73)	0.003
Tobacco use		601 (63.67)	204 (61.82)	0.549
External causes of morbidity and mortality		89 (9.43)	52 (15.76)	0.002
COVID-19		20 (2.12)	5 (1.52)	0.496

T1D: type 1 diabetes. ARHA: alcohol-related hospital admission. Weekend admissions included Saturday or Sunday. ICU: intensive care unit. LOHS: length of hospital stay. IHM: in-hospital mortality. NA: not available. Mental disorders included ICD-10 codes for depression, anxiety, and specific personality disorders (see Table S1). Drug use included ICD-10 codes for mental and behavioral disorders due to the use of opioids, cannabinoids, sedatives or hypnotics, cocaine, and other stimulants, including caffeine, hallucinogens, volatile solvents, and other psychoactive substances (see Table S1). External causes included ICD-10 codes for accidents, injury, and intentional self-harm (see Table S1). *p*-Value for difference by gender calculated with *t*-test (means); Mann–Whitney test (medians) and Fisher exact test (percentages).

**Table 4:** Multivariable analysis of study variables associated with alcohol-related hospital admission (ARHA) in children and young adults with type 1 diabetes in Spain, according to gender (2016−2023).

		Male	Female	Both Gender
Study Variable	Categories	OR (95% CI)	OR (95% CI)	OR (95% CI)
Age groups	12–17 years	Reference	Reference	Reference
	18–23 years	3.12 (2.21–4.42)	1.9 (1.27–2.85)	2.52 (1.93–3.27)
	24–29 years	4.08 (2.9–5.74)	1.32 (0.86–2.01)	2.73 (2.1–3.54)
	30–35 years	5.43 (3.9–7.56)	1.26 (0.83–1.92)	3.37 (2.61–4.35)
Weekend		1.13 (0.96–1.34)	1.32 (1.02–1.71)	1.18 (1.03–1.36)
Diabetic ketoacidosis		1.63 (1.4–1.89)	2.52 (1.97–3.22)	1.84 (1.62–2.09)
Hypoglycemia		1.74 (1.24–2.44)	1.2 (0.67–2.17)	1.55 (1.16–2.07)
Mental disorders		1.22 (0.94–1.57)	1.28 (0.9–1.8)	1.25 (1.02–1.53)
Drug use		6.15 (5.27–7.18)	70.1 (5.32–9.21)	6.40 (5.59–7.33)
Tobacco use		2.71 (2.32–3.18)	4.46 (3.41–5.83)	3.16 (2.76–3.62)
Year of admission	2016	Reference	Reference	Reference
	2017	1.15 (0.85–1.54)	0.99 (0.57–1.73)	1.11 (0.86–1.44)
	2018	1.01 (0.75–1.36)	0.86 (0.49–1.51)	0.97 (0.75–1.27)
	2019	1.06 (0.79–1.43)	1.24 (0.74–2.09)	1.09 (0.85–1.41)
	2020	1.1 (0.82–1.49)	1.17 (0.68–1.98)	1.11 (0.85–1.44)
	2021	1.06 (0.78–1.42)	1.64 (1–2.7)	1.18 (0.92–1.52)
	2022	1.14 (0.85–1.53)	2.14 (1.33–3.45)	1.36 (1.06–1.74)
	2023	0.98 (0.73–1.33)	1.92 (1.18–3.13)	1.18 (0.92–1.52)
Gender	Female	NA	NA	Reference
	Male	NA	NA	2.46 (2.15–2.82)

Weekend admissions included Saturday or Sunday. Mental disorders included ICD-10 codes for depression, anxiety, and specific personality disorders (see Table S1). Drug use included ICD-10 codes for mental and behavioral disorders due to the use of opioids, cannabinoids, sedatives or hypnotics, cocaine, and other stimulants, including caffeine, hallucinogens, volatile solvents, and other psychoactive substances. (See Table S1). OR; Odds Ratio. CI; Confidence Interval. NA; not available.

**Table 5:** Multivariable analysis of study variables associated with admission to the intensive care unit in children and young adults with type 1 diabetes with alcohol-related hospital admission (ARHA) in Spain, according to gender (2016−2023).

		Male	Female	Both Gender
Study Variable	Categories	OR (95% CI)	OR (95% CI)	OR (95% CI)
Age groups	12–17 years	Reference	Reference	Reference
	18–23 years	0.7 (0.31–1.61)	1.65 (0.57–4.73)	0.92 (0.49–1.73)
	24–29 years	0.89 (0.4–1.99)	1.72 (0.58–5.11)	1.15 (0.61–2.17)
	30–35 years	0.7 (0.32–1.53)	3.11 (0.96–9.32)	1.08 (0.58–2.01)
Diabetic ketoacidosis		4.91 (3.12–7.72)	8.47 (3.61–19.86)	5.59 (3.78–8.26)
Hypoglycemia		4.11 (2–8.47)	0.47 (0.05–4.83)	3.28 (1.68–6.4)
Drug use		1.52 (1.25–2.17)	1.78 (1.12–2.42)	1.64 (1.39–1.96)
Year of admission	2016	Reference	Reference	Reference
	2017	1.25 (0.54–2.87)	1.09 (0.23–5.25)	1.24 (0.6–2.56)
	2018	2.9 (1.36–6.19)	2.89 (0.63–13.36)	2.86 (1.47–5.58)
	2019	1.78 (0.81–3.9)	1.59 (0.35–7.19)	1.74 (0.88–3.43)
	2020	1.85 (0.84–4.06)	2.06 (0.45–9.41)	1.87 (0.95–3.71)
	2021	1.44 (0.64–3.22)	1.58 (0.38–6.53)	1.45 (0.73–2.85)
	2022	1.9 (0.87–4.12)	1.71 (0.43–6.81)	1.76 (0.91–3.39)
	2023	1.72 (0.78–3.84)	3.87 (1.03–15.03)	2.08 (1.07–4.09)
Gender	Male	NA	NA	Reference
	Female	NA	NA	1.39 (1.02–1.90)

Drug use included ICD-10 codes for mental and behavioral disorders due to the use of opioids, cannabinoids, sedatives or hypnotics, cocaine, and other stimulants, including caffeine, hallucinogens, volatile solvents, and other psychoactive substances (see Table S1). OR: odds ratio. CI: confidence interval. NA: not available.

## Data Availability

According to the contract signed with the Spanish Ministry of Health and Social Services, which provided access to the databases from the Spanish National Hospital Database, we cannot share the databases with any other investigator, and we have to destroy the databases once the investigation has concluded. Consequently, we cannot upload the databases to any public repository. However, any investigator can apply for access to the databases by filling out the questionnaire available at https://www.sanidad.gob.es/estadEstudios/estadisticas/estadisticas/estMinisterio/SolicitudCMBDdocs/Formulario_Peticion_Datos_RAE_CMBD.pdf (accessed on 5 June 2025). All other relevant data are included in this paper.

## Supplementary Materials

The following supporting information can be downloaded at https://www.mdpi.com/article/10.3390/jcm14124053/s1, Table S1: Diagnosis analyzed with the corresponding ICD-10 codes; Table S2: Clinical characteristics and hospital outcomes (ARHA) in children and young adults with type 1 diabetes, with and without alcohol-related hospital admission in Spain according to gender (2016−2023).

## Abbreviations

The following abbreviations are used in this manuscript:

ER	emergency room
T1D	type 1 diabetes
DKA	diabetic ketoacidosis
ARHAs	alcohol-related hospital admissions
SHDD	Spanish Hospital Discharge Database
SMH	Spanish Ministry of Health
ICU	intensive care unit
ICD-10	International Classification of Diseases, Tenth Revision
T2D	type 2 diabetes
LOHS	length of hospital stay
IHM	in-hospital mortality
