# Supplementary material for: Alcohol-Related Hospitalizations Among Adolescents and Young Adults with Type 1 Diabetes in Spain, 2016−2023
Source: J Clin Med. 2025 Jun 8;14(12):4053. doi: 10.3390/jcm14124053 (PMC12194657; doi:10.3390/jcm14124053)
Supplement: Supplementary file 1 [file jcm-14-04053-s001.zip › jcm-3684456-supplementary.pdf]

Table S1. Diagnosis analyzed with their corresponding ICD10 codes.

**CONDITION**	**ICD 10 CODES**
Type 1 diabetes	E10
Alcohol-associated hospitalizations	E24.4; E51.2; F10; G31.2; G62.1; G72.1; I42.6; K29.20; K29.21 K70; K85.2; K86.0; T51.0; O35.4; R78.0; Y90; Z71.4
Diabetic ketoacidosis	E10.10, E10.11
Hypoglycemia	E10.641, E10.649
Mental disorders	F32, F33, F40, F41, F60
Drug use	F11, F12, F13, F14, F15, F16, F18, F19
Tobacco use	F17, Z72.0, Z87.891, T65.2
External causes of morbidity and mortality	S00 to T14.8x, T14.90, V00 to X58, X60-X84
COVID 19	U071

Table S2. Clinical characteristics and hospital outcomes (ARHA) in children and young adults with type 1 diabetes, with and without alcohol-related hospital admission in Spain according to gender (2016-2023)

	BOTH GENDER			MALE			FEMALE		
	Not ARHA	ARHA	p	Not ARHA	ARHA	p	Not ARHA	ARHA	p
Age Mean (SD)	23.31(7.5)	27.22(5.95)	<0.001	22.75(7.57)	27.88(5.66)	<0.001	23.8(7.4)	25.31(6.34)	<0.001
12-17 years, n (%)	11153(29.49)	78(6.12)	<0.001	5775(32.83)	43(4.56)	<0.001	5378(26.59)	35(10.61)	<0.001
18-23 years, n (%)	8074(21.35)	300(23.55)		3761(21.38)	191(20.23)		4313(21.32)	109(33.03)	
24-29 years, n (%)	8015(21.19)	352(27.63)		3477(19.77)	262(27.75)		4538(22.43)	90(27.27)	
30-35 years, n (%)	10575(27.96)	544(42.7)		4576(26.02)	448(47.46)		5999(29.66)	96(29.09)	
Weekend, n (%)	7785(20.59)	333(26.14)	<0.001	3717(21.13)	242(25.64)	<0.001	4068(20.11)	91(27.58)	<0.001
Admission to ICU, n (%)	4513(11.93)	265(20.8)	<0.001	1993(11.33)	181(19.17)	<0.001	2520(12.46)	84(25.45)	<0.001
LOHS Median (IQR)	4(4)	4(4)	0.857	4(4)	3(4)	0.609	4(4)	4(4)	0.983
IHM , n (%)	107(0.28)	1(0.08)	0.171	56(0.32)	1(0.11)	0.251	51(0.25)	0(0)	0.361
Diabetic ketoacidosis, n (%)	15727(41.59)	771(60.52)	<0.001	7797(44.33)	552(58.47)	<0.001	7930(39.2)	219(66.36)	<0.001
Hypoglycemia, n (%)	1589(4.2)	60(4.71)	0.375	646(3.67)	47(4.98)	0.039	943(4.66)	13(3.94)	0.536
Mental disorders, n (%)	2462(6.51)	197(15.46)	<0.001	744(4.23)	113(11.97)	<0.001	1718(8.49)	84(25.45)	<0.001
Drug use, n (%)	2068(5.47)	633(49.69)	<0.001	1338(7.61)	492(52.12)	<0.001	730(3.61)	141(42.73)	<0.001
Tobacco use, n (%)	6303(16.67)	805(63.19)	<0.001	3379(19.21)	601(63.67)	<0.001	2924(14.46)	204(61.82)	<0.001
External causes of morbidity and mortality, n (%)	2122(5.61)	141(11.07)	<0.001	1113(6.33)	89(9.43)	<0.001	1009(4.99)	52(15.76)	<0.001
Covid 19, n (%)	626(1.66)	25(1.96)	0.401	313(1.78)	20(2.12)	0.445	313(1.55)	5(1.52)	0.962
2016, n (%)	4687(12.39)	119(9.34)	<0.001	2165(12.31)	93(9.85)	0.247	2522(12.47)	26(7.88)	<0.001
2017, n (%)	4574(12.1)	144(11.3)		2063(11.73)	116(12.29)		2511(12.41)	28(8.48)	
2018, n (%)	4799(12.69)	139(10.91)		2162(12.29)	112(11.86)		2637(13.04)	27(8.18)	
2019, n (%)	5047(13.35)	163(12.79)		2342(13.32)	126(13.35)		2705(13.37)	37(11.21)	
2020, n (%)	4446(11.76)	150(11.77)		2160(12.28)	115(12.18)		2286(11.3)	35(10.61)	
2021, n (%)	4689(12.4)	171(13.42)		2153(12.24)	121(12.82)		2536(12.54)	50(15.15)	
2022, n (%)	4753(12.57)	211(16.56)		2234(12.7)	142(15.04)		2519(12.45)	69(20.91)	
2023, n (%)	4822(12.75)	177(13.89)		2310(13.13)	119(12.61)		2512(12.42)	58(17.58)	

T1D; type-1 diabetes ARHA; Alcohol-related hospital admission. Weekend admissions included Saturday or Sunday. ICU. Intensive Care Unit. LOHS: Length of hospital stay. IHM; In Hospital Mortality. NA; not available. Mental disorders included ICD 10 codes for depression, anxiety and specific personality disorders (See table S1). Drug use included ICD 10 codes for Mental and behavioral disorders due to the use of opioids, cannabinoids, sedatives or hypnotics, cocaine, other stimulants, including caffeine, hallucinogens, volatile solvents and other psychoactive substances. (See table S1).External causes included ICD 10 codes for accidents, Injury and Intentional self-harm (See table S1). P value for difference by ARHA calculated with t test (means); Mann-Whitney test (medians) and Fisher exact test (percentages).
